# Inhibition of Aquaporin 4 Decreases Amyloid Aβ40 Drainage Around Cerebral Vessels

**DOI:** 10.1007/s12035-020-02044-8

**Published:** 2020-08-11

**Authors:** Gabriela-Camelia Rosu, Bogdan Catalin, Tudor Adrian Balseanu, Mogoanta Laurentiu, Margaritescu Claudiu, Samir Kumar-Singh, Pirici Daniel

**Affiliations:** 1 Department of Research Methodology, University of Medicine and Pharmacy of Craiova, Petru Rares Street 2, 200349 Craiova, Dolj Romania; 2 Experimental Research Centre for Normal and Pathological Aging, University of Medicine and Pharmacy of Craiova, Craiova, Romania; 3 Department of Physiology, University of Medicine and Pharmacy of Craiova, Petru Rares Street 2, 200349 Craiova, Dolj Romania; 4 Department of Histology, University of Medicine and Pharmacy of Craiova, Craiova, Romania; 5 Department of Pathology, University of Medicine and Pharmacy of Craiova, Craiova, Romania; 6 Faculty of Medical & Health Sciences, Molecular Pathology Group, Laboratory of Cell Biology & Histology, University of Antwerp, Antwerp, Belgium

**Keywords:** Aquaporin 4, Amyloid, TGN-020, Aβ40, Perivascular drainage, Alzheimer’s disease

## Abstract

Aquaporin-4 (AQP4) is located mainly in the astrocytic end-feet around cerebral blood vessels and regulates ion and water homeostasis in the brain. While deletion of AQP4 is shown to reduce amyloid-β (Aβ) clearance and exacerbate Aβ peptide accumulation in plaques and vessels of Alzheimer’s disease mouse models, the mechanism and clearing pathways involved are debated. Here, we investigated how inhibiting the function of AQP4 in healthy male C57BL/6 J mice impacts clearance of Aβ40, the more soluble Aβ isoform. Using two-photon in vivo imaging and visualizing vessels with Sulfurodamine 101 (SR101), we first showed that Aβ40 injected as a ≤ 0.5-μl volume in the cerebral cortex diffused rapidly in parenchyma and accumulated around blood vessels. In animals treated with the AQP4 inhibitor TGN-020, the perivascular Aβ40 accumulation was significantly (*P* < 0.001) intensified by involving four times more vessels, thus suggesting a generalized clearance defect associated with vessels. Increasing the injecting volume to ≥ 0.5 ≤ 1 μl decreased the difference of Aβ40-positive vessels observed in non-treated and AQP4 inhibitor-treated animals, although the difference was still significant (*P* = 0.001), suggesting that larger injection volumes could overwhelm intramural vascular clearance mechanisms. While both small and large vessels accumulated Aβ40, for the ≤ 0.5-μl volume group, the average diameter of the Aβ40-positive vessels tended to be larger in control animals compared with TGN-020-treated animals, although the difference was non-significant (*P* = 0.066). Using histopathology and ultrastructural microscopy, no vascular structural change was observed after a single massive dose of TGN-020. These data suggest that AQP4 deficiency is directly involved in impaired Aβ brain clearance via the peri-/para-vascular routes, and AQP4-mediated vascular clearance might counteract blood-brain barrier abnormalities and age-related vascular amyloidopathy.

**Electronic supplementary material:**

The online version of this article (10.1007/s12035-020-02044-8) contains supplementary material, which is available to authorized users.

## Introduction

The histopathological hallmark of Alzheimer disease (AD) is represented by the accumulation of amyloid β-peptide (Aβ) plaques and neurofibrillary tangles generated by abnormally phosphorylated tau protein in the cortex of patients and is significantly associated with neurovascular dysfunction and neuronal loss [1, 2]. Genetic investigations have placed Aβ upregulation, either as normal or as modified less soluble and more toxic isoforms, as a key factor in AD pathogenesis. Genetically determined AD is in fact rare [3, 4], and sporadic late onset cases (LOAD) account for the majority of patients, suggesting that an impaired Aβ degradation/clearance due to aging and less efficient catabolic/drainage pathways might be more central to the disease rather than Aβ overproduction. While LOAD is classically seen as an end-of-life pathology, careful epidemiological analysis suggests that young and/or middle life exposer to certain risk factors, namely, activity and cardiovascular performance, poor diet with major emphasis on insulin resistance, and even depression, impacts patient outcome and might lead to an earlier onset of disease [5–7]. This raises more questions about the exact mechanism by which Aβ causes AD with several competing theories currently being researched, such as a direct toxicity of Aβ and its isoforms [8–10], a pro-oxidative stress shift in the brain [11, 12], a direct receptor interference, and mitochondrial dysfunction—all leading to synaptic dysfunction and neuronal loss [10, 13, 14]. Interestingly, the soluble (Aβ40) and insoluble (Aβ42) fractions have been linked to different pathological events. While high levels of the soluble Aβ isoform precipitate in the blood vessels walls as cerebral amyloid angiopathy (CAA) and directly contribute to the cognitive decline [15, 16], Aβ42 is the main component of the senile plaques found in AD [17]. Regardless of the cause, Aβ production/clearance is still imbalanced in AD patients. With up to 50% of the Aβ being cleared across the blood-brain barrier (BBB) [18], it seems that investigating the mechanism by which Aβ is cleared needs a more careful consideration, especially in respect to Aβ40 and Aβ42 isoforms. In this respect, Aβ40 is the major isoform shown to be cleared through and along vascular route (reviewed in [19]).

The most abundant water diffusion channel in the CNS is aquaporin 4 (AQP4), expressed mostly on the membranes of ependymal cells, perivascular and subpial astrocytes, and under normal circumstances; the expression is highly polarized to astrocytic end-feet which come in contact with the vascular basement membranes as part of the BBB [20]. The water-exchange around the BBB has been showed to be highly dependent on AQP4, both in normal and pathological conditions. Thus, AQP4 deletion in mice results in an almost one-third decrease of the brain water uptake in normal conditions [21], and inducing cytotoxic brain oedema with an intact BBB (like water intoxication and an ischemic stroke without hemorrhagic transformation) results in significantly decreased brain oedema compared with wild animals [22, 23]. As expected, vasogenic oedema, like in the case of intraparenchymal fluid infusion, cortical-freeze injury, brain tumors, brain abscess, and subarachnoid hemorrhages, on the other hand, leads to increased brain water retention in AQP4^−/−^ animals compared with their non-transgenic counterparts [24, 25]. Not only AQP4 knock-out leads to reduced cytotoxic oedema after an ischemic event but also singular administration of the TGN-020 AQP4 inhibitor, both 30 min before and 15 min after the ischemia [26, 27]. We and others have showed that in ischemic conditions, like after a stroke with non-hemorrhagic transformation, AQP4 loses its polarization for the end-feet and becomes expressed all over the astrocytic membrane not noly in rats but also in humans [27–29].

Recently, with the viewpoint to elucidate the mechanisms of cerebral water exchange and interstitial fluid drainage in the brain [27, 30, 31], AQP4 has been also putatively linked with AD [32, 33]. This is based on work done on transgenic animals overexpressing Aβ and lacking AQP4 channel [34–36]. However, in these situations, Aβ is pathologically overproduced that overwhelms clearance pathways [37]. To understand the role of AQP4 in perivascular clearance of Aβ under physiological conditions mimicking early prodromal stages of AD, we evaluated here the drainage of soluble Aβ40 after a transient inhibition of AQP4.

## Materials and Methods

### Animals and Treatment

The study was performed on 26 male C57BL/6 J (*N* = 8 for ex vivo studies and *N* = 18 for in vivo studies) mice aged 3–4 months [mean weight 25.5 g; standard deviation (SD) 1.84 g]. The animals were housed in a controlled 12 h/12 h of light/dark cycle, with unlimited access to water and food. The night prior to the beginning of experiments, food was withdrawn from the cages. All experiments have been conducted following the Federation of European Laboratory Animal Science Associations (FELASA) guidelines and have been approved by the local Ethics Committee of the University of Medicine and Pharmacy of Craiova (no 91/13.09.2018), under the Romanian and European laws, in accordance with Helsinki ethical guidelines.

Given the fact that there is no study yet to address the effect of TGN-020 as a function of its concentration, and all previous pilot studies available used maximal dosages, together with the fact that, to our knowledge, it has no histopathological or clinical side effects, for all experiments, a single massive dose of TGN-020 AQP4 inhibitor (400 mg/kg) was used, in order to saturate the AQP4 and identify any putative subtle changes across the brain-blood barrier. The TGN-020 chloride salt [N-(1,3,4-thiadiazol-2-yl) pyridine-3-carboxamide dihydrochloride, MW = 279.1463] (Ukrorgsyntez Ltd., Kiev, Ukraine) was dissolved in 0.4 ml sterile distillate water and titrated with 2 M NaOH to a pH of 8. In order to induce, as much as possible, the same volumetric and osmotic changes in all animals, and given the untitered final concentration of the TGN-020 chloride salt as roughly 0.1 mol/l, control animals received 0.4 ml of isotonic saline (0.15 mol/l) [27]. At 30 min after the IP injections, animals designated to the ex vivo histopathology and EM arm of the study (TGN-020 *N* = 5 and Control *N* = 3) were anesthetized and perfused first with PBS (5 min) and then with a mixture of 3.4% Neutral Buffered Formalin (NBF) and 3% Glutaraldehyde, pH 7.2 (10 min), after which brains dissected. One hemisphere was kept in NBF (neutral buffered-formalin)/glutaraldehyde for EM analysis, and the other half was placed in 4% NBF for 24 h and further processed for paraffin embedding and histopathology. The animals to be utilized for the in vivo analysis (*N* = 18) received the same IP regimens of TGN-020 salt or isotonic saline after experimental setup and stereotaxic fixation, at 30 min prior to the Aβ injection.

### Amyloid Injection, Two-Photon Laser Scanning Microscopy, and Fluorescence Microscopy

High-resolution, time-lapse in vivo imaging was performed using a 7MP Zeiss two-photon laser scanning microscope (2P-LSM) (Carl Zeiss MicroImaging GmbH, Jena, Germany) on anesthetized (120 mg/kg ketamine and 12 mg/kg xylazine) C57BL/6J mice (*N* = 18). Prior to the imaging session, a cranial window was implanted, using a previously described method [38]. In summary, after the skin and soft tissue were removed, a custom-made handler was fixed to the right parietal skull of the animals using dental cement. A small craniotomy was performed, right over the somatosensory cortex. After any bleeding was stopped, a 20-min 2P-LSM session was used as baseline. Before starting the imaging session, optimization was made by placing the animal on a special custom-made imaging table, capable of manual tilting the animal in *x* and *y* axes [39]. 2P-LSM imaging was performed in between 100 and 200 μm under the surface, in Z-stack planes of 425.10 × 425.10 μm, using a W-Plan Apochromat 20×/1.0 DIC Vis-IR water immersion objective (Carl Zeiss) controlled by ZEN 2010 imaging software (Carl Zeiss). The fluorophore was excited using an fs-pulsed titanium-sapphire laser (Chameleon Vision II, Coherent, Glasgow, UK) with a peak power of 3.5 W tuned to 910 nm [38]. Using a front filling technique, a glass pipet, with an opening of 15–25 μm, was filled with either 0.5 or 1 μl human amyloid-β 1–40 peptide (Hilyte Fluor 488 labelled, AnaSpec EGT, Fremont, CA, USA catalog number AS-60491-01), diluted as 0.5 mg/ml (100 μM in 0.25% NH4OH, and frozen at − 30 °C as 20 μl aliquots) [40]. Before loading the pipet, thawed diluted aliquots were continuously mixed with a 20-μl pipette in order to break apart any preformed aggregates, and any remaining material was discarded. The pipette was inserted into the somatosensorial cortex utilizing a micromanipulator under transmission light microscopy settings and a × 5 objective, taking special care to avoid piercing local blood vessels. The content of the pipette was slowly injected (~ 1 min) utilizing an injection pump, under microscope feedback for the > 0.5-μl group (*N* = 5 controls and *N* = 5 treated with TGN-020), while for the < 0.5-μl volume (*N* = 4 controls and *N* = 4 treated with TGN-020), the pump was stopped and momentarily reversed immediately after the first puff was observed. This process utilized a custom-made 3D-printed and electronically controlled pump that advanced the piston of a 1-μl Hamilton syringe [41]; the needle of the syringe was hydraulically connected with the injection pipette utilizing silicone gun oil and inextensible Teflon tubing. Sulfurodamine 101 (Merck KGaA, Darmstadt, Germany) was dissolved in saline to obtain a 100 μM solution. To visualize blood vessels under the 2P-LSM, 0.2 ml of the prepared solution was injected IP 5 min prior to the imaging sessions. Animals were kept under anesthesia, and for the following 40 min, scanning sessions were initiated at each 5 min. At the end of the experiment, animals were administrated one more anesthetic dose, then they were decapitated, brains removed, and cut as 30-μm thick sections using a vibratome. Sections were collected on poly-L-lysine (PLL)-coated slides, fixed in 4% NBF for 1 min and then a cover slipped with Vectashield H-1200 (Vector Laboratories, Peterborough, UK) mounting medium.

Conventional fluorescent images were grabbed with a × 10 plan apochromat objective (NA = 0.30), utilizing a Nikon Eclipse 90i motorized microscope (Nikon Instruments Europe BV, Amsterdam, The Netherlands) equipped with a Prior OptiScan ES111 motorized stage (Prior Scientific, Cambridge, UK), single-band fluorescence filters, a 16-megapixel Nikon DS-Ri-2 CMOS cooled camera (Nikon), and driven by the Nikon NIS-Elements AR image analysis software.

### Fluorescence Microscopy and Post-fixation Basement Membrane Evaluation

Brain hemispheres fixed in NBF were further processed for paraffin embedding, and 4-μm thick sections were cut on a microtome and collected on PLL-coated slides. Besides classical hematoxylin-eosin staining, the tissue was processed for immunohistochemistry for visualizing endogenous immunoglobulins and vascular basement membranes (BM). For BM/astrocyte visualization, the sections were first digested with proteinase K for 20 min at 37 °C (Dako, Glostrup, Denmark), unspecific binding sites were blocked with 3% skimmed milk (Bio-Rad Laboratories GmbH, München, Germany), then the primary antibodies [rabbit-anti-laminin, 1:100 (Abcam, Cambridge, UK) and chicken anti-glial fibrillary acidic protein (GFAP), 1:500 (Novus Biologicals, Centennial, CO, USA)] were added together for 18 h at 4 °C and visualized with a mixture of goat anti-rabbit Alexa 596/goat anti-chicken Alexa 488 secondary antibodies (1:300, Thermo Fisher Scientific, Waltham, MA, USA) after 1-h incubation at room temperature. The slides were cover slipped with a DAPI-containing mounting medium (Vectashield, H-1200, Vector Laboratories) and protected with nail polish. For endogenous mouse immunoglobulin visualization, the slides were blocked with 3% skimmed milk, and then an anti-mouse horse-radish peroxidase (HRP)-labelled polymer was added on the slides for 1 h (goat anti-mouse HRP, Vector Laboratories). After thorough washing, the signal was visualized with Tyramide Alexa 488 diluted as 1:200 dilution in amplification medium, for 15 min (Thermo Fischer).

Slides were visualized, and random images were captured with the Nikon Eclipse 90i microscope utilizing a × 60 plan apochromat immersion objective (NA = 1.40). All images were saved in Nikon’s proprietary format and processed first by a 5-iteration blind deconvolution algorithm (NIS Elements AR), then for all blood vessels in each image, the thickness of the basement membranes was measured with a direct measuring tool in NIS-Elements. Only external (abluminal) basement membranes have been considered and measured in their thickest region. Perivascular spaces have not been considered, and vessels with unclear profiles were also not considered. Measurements have been averaged for each slide and then for each brain region of each animal.

### Electron Microscopy

Designated brain hemispheres were fixed in the NBF/Glutaraldehyde mixture, and then representative sections of grey matter were microdissected and processed for TEM according to MacGregor Sharp et al. [42]. Briefly, tissue blocks were trimmed and 80-nm ultrathin sections cut using an Ultracut E microtome. Grids were examined using a Hitachi HT7700 transmission electron microscope operating a Morada G3 digital camera and Radius image capture software (EMSIS, Münster, Germany). Transmission electron microscopy (TEM) was performed by methodically scanning each sample from top right to bottom left. High-resolution low power images of 20 capillaries from the grey matter of each mouse (120 total) were digitally photographed.

### Data Analysis

For the detailed analysis of in vivo Aβ drainage and histopathology data, parameters like vessel positivity for Aβ, vessel diameters, Aβ colocalization, and BM thickness were determined by direct manual counting and measurements in ZEN 2010 and Image ProPlus 7 packages. For in vivo analysis and histopathology measurements, data were averaged first per animal, then per treatment group (treated/untreated), followed by statistical analysis. We defined a vessel as being Aβ-positive when either the red SR 101 signal colocalized with the green Aβ or when there was an unambiguous compaction of green Aβ signal directly on the vessel wall. A proof-of principle study was also conducted to assess if an image analysis algorithm might be able to accurately differentiate these vessels in our analysis (See Supplementary Material and Supplementary Fig. 1**).** All data were analyzed using GraphPadPrism 7 and Microsoft Excel. For statistical comparison of two groups, a Student *t* test was used, while for multiple comparisons, we utilized a Tukey corrected two-way ANOVA. For EM images, Adobe Photoshop CS6 (Adobe Inc., San Jose, CA, USA) was used to perform a detailed analysis of capillary wall composition. Briefly, the endothelium, intramural cells, and basement membrane were segmented and analyzed for percentage surface area of the vessel wall using inbuilt Adobe Photoshop measure and analysis tools. Statistical analysis was performed using SPSS and univariate analysis of variance allowing for multiple comparisons (significance set at *P* < 0.05). All figures display mean value and standard deviation of the means (SD). The statistical significance is displayed as follows: *: *P* < 0.05, **: *P* < 0.01, and ***: *P* < 0.001.

## Results

### Vascular Intramural Aβ Drainage Is Dependent on the Functionality of AQP4 Channels

**Fig. 1:**
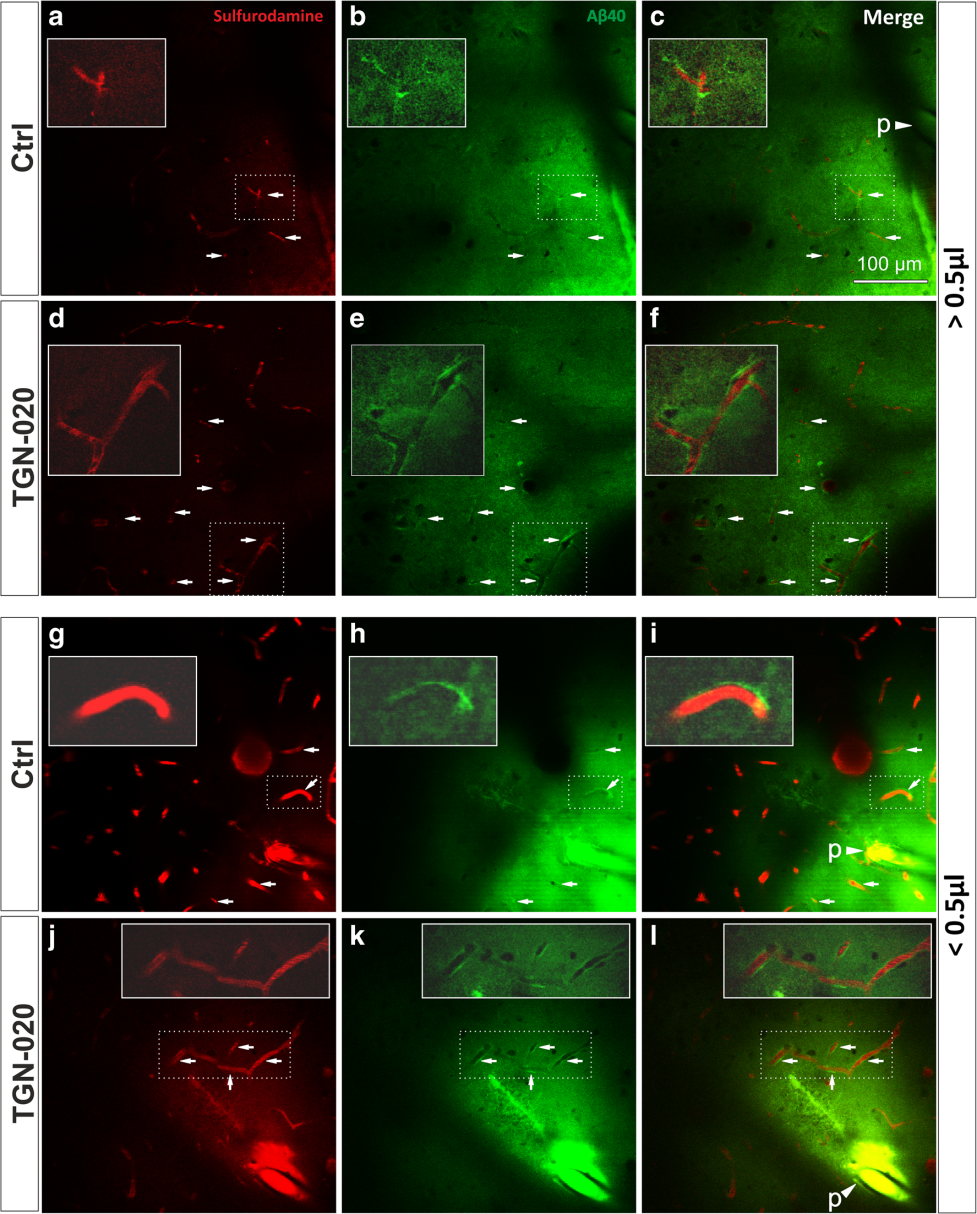
Representative two-photon microscopy images of anesthetized mice, 10 min after Aβ40-A488 was injected in the upper layers of the right somatosensory cortex. Injecting a large volume floods the water drainage system, leading to some Aβ40 deposits around blood vessels in control animals (**a**–**c**). The number of vessels surrounded by Aβ40 is greater when blocking AQP4 function (**d**–**f**). Injecting small volumes of Aβ40 leads to a limited number of vessels exhibiting Aβ40 deposits in control animals (**g**–**i**); however, blocking AQP4 channels generates multiple Aβ40 accumulation around vessels (**j**–**l**). In each image, insets represent magnified exemplary areas demonstrating abluminal Aβ accumulation. Arrows point to regions of interest with clear-cut Aβ40 being retained around the vascular lumens; arrow heads indicating a “p” denominate the position of the injection pipette tip, filled with the fluorescent Aβ40

**Fig. 2:**
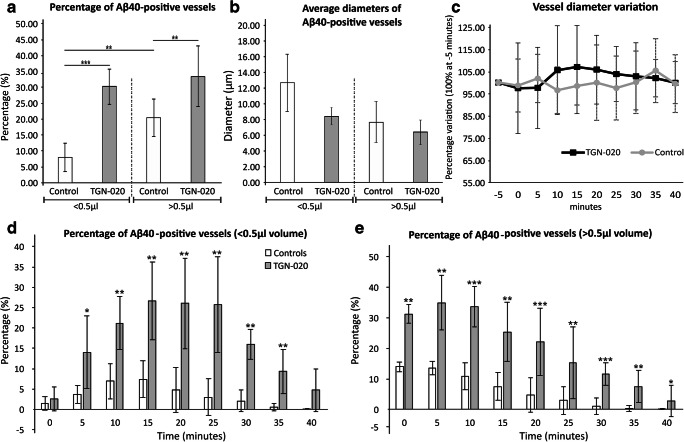
Systemic administration of AQP4 inhibitor reveals a higher number of blood vessels showing Aβ40 deposits. **a** The total volume injected impacts the drainage system; however, regardless of the injected volume, blocking AQP4 channels leads to an increase frequency of Aβ40 deposits around vessels, compared with controls, predominantly for vessels (**b**) with a smaller diameter. **c** When analyzing the variation over time of the blood vessel diameters, no change could be observed. Investigating the dynamic of Aβ40 drainage around the injected site, we could see differences for both small (**d**) and large (**e**) injected volumes. The average number of elements per animal was considered for analysis in all instances. Mean ± SD; ^∗^*P* < 0.05, ^∗∗^*P* < 0.01, ^∗∗∗^*P* < 0.001; *N* = 9 control animals (4 for < 0.5 μl; 5 for > 0.5 μl) and 9 TGN-020 animals (4 for < 0.5 μl; 5 for > 0.5 μl). For **a**, an average of *n* = 51 ± 9.16/43 ± 11.83 vessels have been considered for control group (< 0.5 μl/> 0.5 μl), and respectively *n* = 46.83 ± 9.57/53.83 ± 16.84 vessels have been considered for the TGN-020 group (< 0.5 μl/> 0.5 μl)

We first aimed to evaluate the diffusion and vascular intramural drainage of soluble Aβ in the brain of the living animals. For this, we injected AlexaFluor488-labelled soluble full-length Aβ40 peptide into the superficial cortex of C57BL/6J mice and followed the injection site under the 2P-LSM for 45 min. During the experiment, blood vessels were visible after systemic administration of SR 101. In vivo imaging confirmed that Aβ40 rapidly diffused into the surrounding neuropil regardless of the injected volume (< 0.5 μl or > 0.5 μl). In all experiments, control animals displayed accumulation of Aβ40 peptide around blood vessels near the injection site within the first minute of the injection, with some being visible for up to 40 min afterwards (Fig. 1 and Supplementary Fig. 2). After systemically injecting TGN-020 AQP4 inhibitor prior to Aβ40 injection, in vivo imaging clearly showed more vascular accumulation of Aβ40 peptide compared with the control group, for both injected volumes (Fig. 1). A proof-of principle analysis also revealed that the Aβ40 signal densification in the close proximity of the vessels needed to deem the vessel positive can also be differentiated from negative vessels by an intensity-profile algorithm (Supplementary Fig. 1). Counting Aβ40-positive vessels revealed that their number had almost increased four times for TGN-020-injected animals (30.221 ± 5.524%) for less than 0.5-μl injected volumes compared with controls (7.946 ± 4.458%) (*P* < 0.001) (Fig. 2, a). Injecting a volume of more than 0.5 μl drastically increased the number of vessels in both control animals (20.393 ± 5.857%) and the TGN-020-treated animals (33.475 ± 9.624%), maintaining a significant difference between the control and treated animal groups (*P* = 0.001).

Next, we evaluated if the drainage of Aβ occurs uniformly for vessels of all sizes. Measuring the diameter of the vessels showing Aβ deposits, we showed that for the small volume injections, Aβ accumulation was greater around smaller vessels in treated animals (8.506 ± 1.081 μm) compared with controls (12.795 ± 3.635 μm), although the difference did not attain statistical significance (Fig. 2, b, *P* = 0.066). For the higher volume injections, the diameters were much more homogenous in treated animals (6.550 ± 1.571 μm) and controls (7.755 ± 2.629; *P =* 0.896). For vessels not showing Aβ deposits around them, no difference between their average diameters for AQP4-treated (5.563 ± 0.632 μm) and control animals (5.092 ± 1.085 μm; *P* = 0.27). During the course of the experiment, blood vessel diameter varied slightly over time (*P* > 0.05), probably as a direct result of pressure variations and pulsatility (Fig. 2, c).

Although the AQP4 inhibitor was given systemically, we further investigated if decreasing Aβ concentration from the injection point influenced Aβ disposition around vessels. Counting vessels as a function of distance from the tip of the injecting pipet, we were not able to identify any difference between control and AQP4 inhibitor group, with vessels depositing Aβ both near (within 20 μm) and distant to the injection site (at more than 360 μm away from the tip) (*P* = 0.32).

Next, we studied the time dynamic of vascular Aβ clearance by analyzing the persistence of the perivascular Aβ around the injection site (Fig. 2, d, e). For the small injection volume, the percentage of positive vessels slowly increased for both TGN-020-treated and control animals over the first 15 min, after which it plateaued for 10 min, and then steeply decreased over the next 15 min when the experiments were stopped (Fig. 2, d). Between 5 and 35 min, there was a significantly higher number of positive vessels for the treated group compared with the control animals (*P*, at least, < 0.05). For the higher volume injection, the number of positive vessels increased more rapidly and then slowly decreased until the end of the follow-up (Fig. 2, e). Here, basically, at every point of investigation, TGN-020-treated animals showed significantly more vessels accumulating Aβ (*P*, at least, < 0.05).

### Ex Vivo Fluorescence Imaging Confirms the In Vivo Vascular Intramural Aβ40 Disposition

**Fig. 3:**
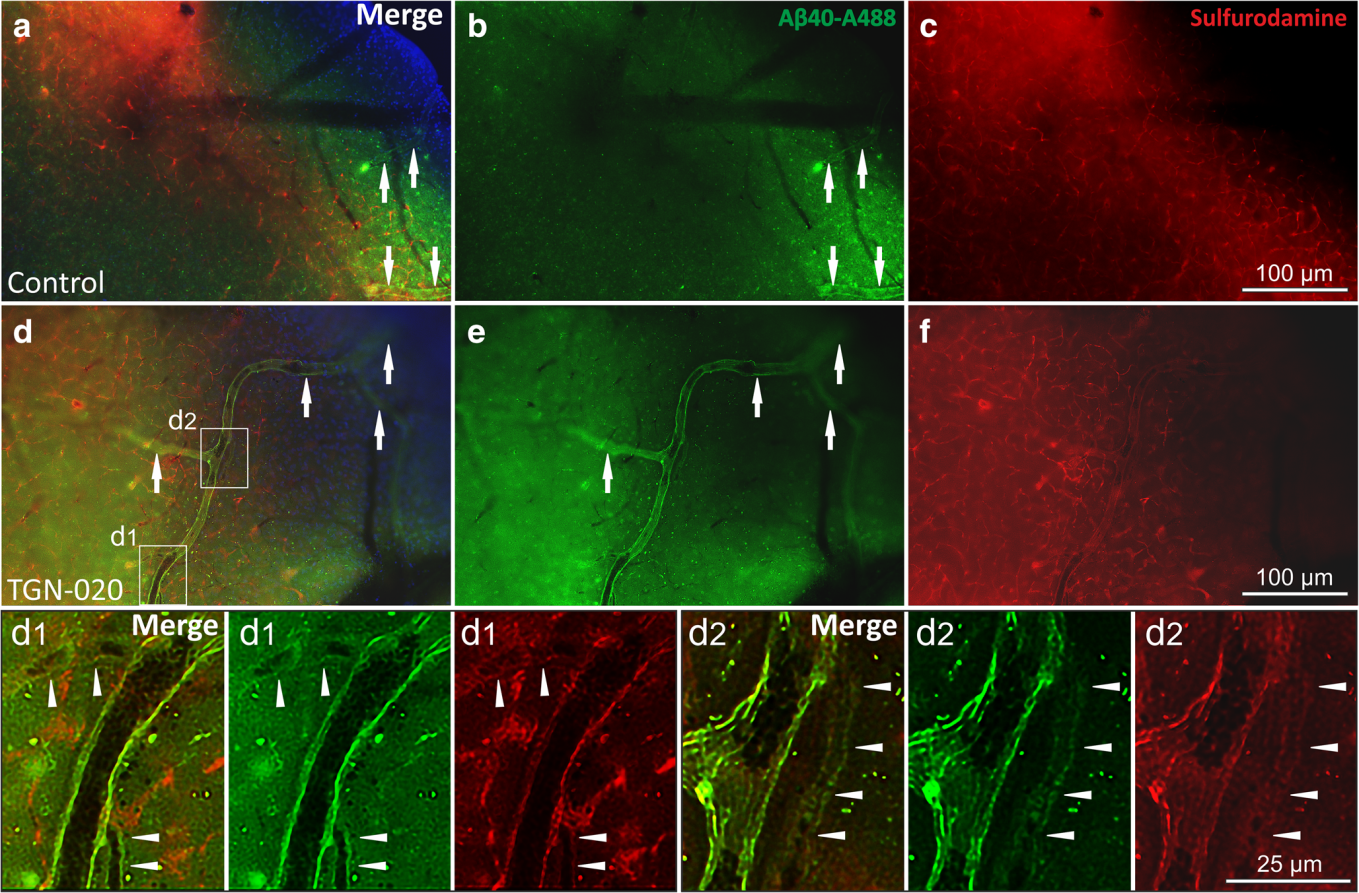
Ex vivo fluorescence imaging of Aβ40 and Sulfurodamine 101 in control (**a**–**c**) and TGN-020-treated animals (**d**–**f**, d1, d2 insets). Colocalization of the two signals is still visible after tissue processing in more vessels for treated animals (arrows) and also in more numerous smaller vessels (arrowheads)

On rapidly prepared vibratome cortical slices from the experimental animals, we could still identify fluorescent Aβ40 and SR 101 in the blood vessels within 60 min after Aβ injection **(**Fig. 3). In control animals, Aβ40 could be identified mostly diffusing not only in the parenchyma but also in the walls of some larger penetrant blood vessels (Fig. 3, a–c). Compared with in vivo imaging, SR 101 was washed away from within the vascular lumens, however, still intimately coated the endothelia (Fig. 3, a, c, d, f). Accordingly, there was still a high degree of colocalization between Aβ and SR 101 in the vessel walls. When comparing controls with TGN-020-treated animals, not only did blood vessels overall retained more Aβ40 in their walls but in treated animals, we could also identify smaller diameter vessels retaining the dye, thus confirming the in vivo observation of significantly increased vascular intramural accumulation in AQP4 inhibitor-treated animals (Fig. 3, d–f). Aβ signal that was still visible in the blood vessels walls showed a perfect colocalization with the remaining SR 101, at the level of clearly identifiable vessel walls folds.

### TGN-020-Treated Animals Show a Tendency for Reduced Vascular Permeability for Immunoglobulins

**Fig. 4:**
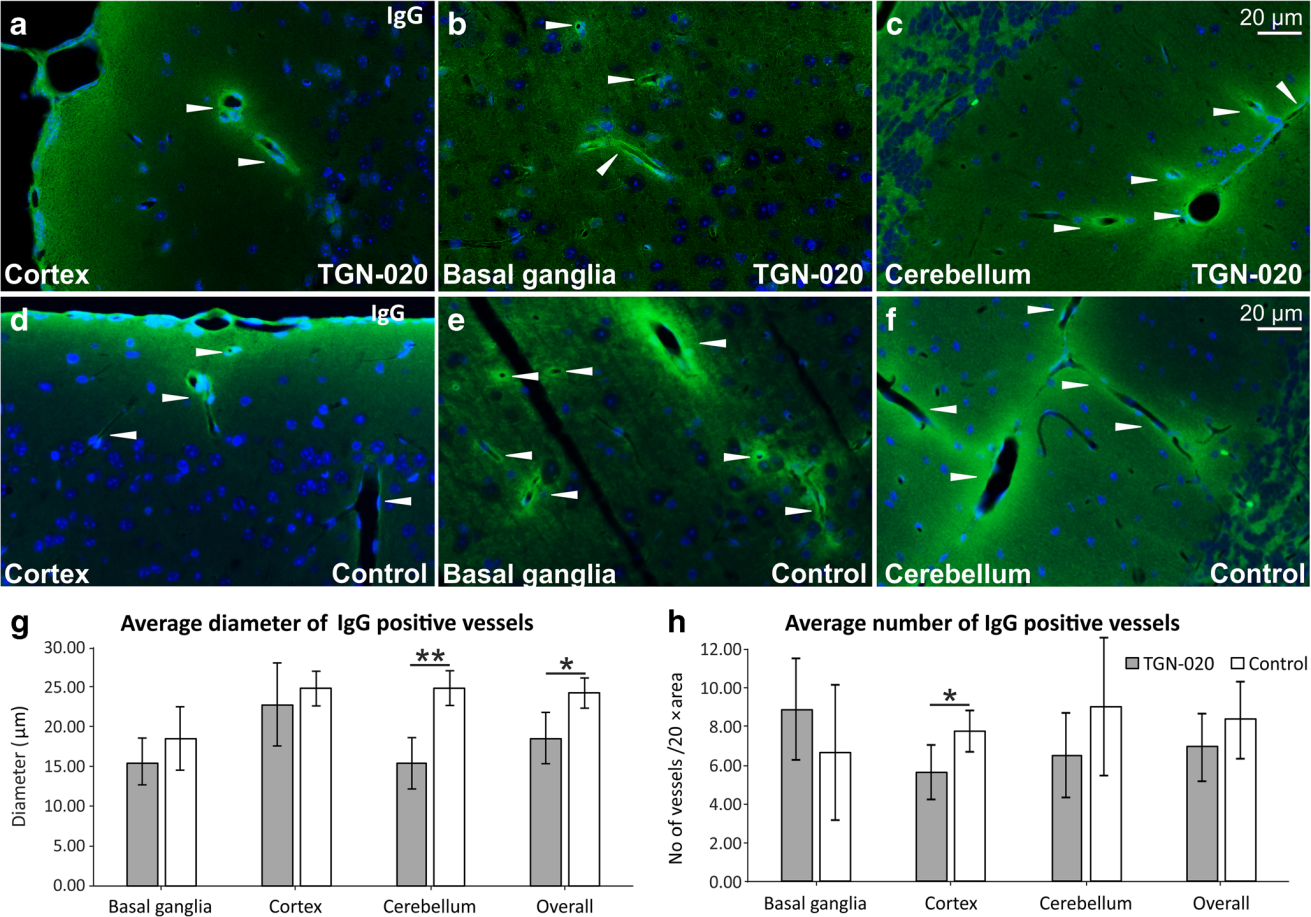
Post-fixed tissues immunostained for endogenous murine immunoglobulins (**a**–**f**) show only a regional decrease in IgG permeability for TGN-020-treated animals, both as the average diameter of positive vessels (**g**) and as the number of positive vessels (**h**). The average value per animal was considered for analysis. Mean ± SD; ^∗^*P* < 0.05, ^∗∗^*P* < 0.01. *N* = 5 animals for the TGN-020 group and 3 animals for the control group

We did not observe any gross morphological difference between the blood vessels of TGN-020-treated and control animals as visualized on the paraffin-embedded hemispheres, and where no acute bleedings or any inflammatory infiltrate abutting the vessel walls. We further studied if there were any immediate change in the BBB permeability following the high dosage of TGN-020. To this end, a staining for endogenous mouse immunoglobulins was performed, which revealed occasional vascular staining involving the vessel wall itself but also with a diffuse staining pattern in the immediate surrounding neuropil (Fig. 4, a–f). This pattern was present in all cortical and subcortical regions, including the cerebellum. In order to assess putative differences between the two animal groups, we calculated the average diameters of IgG-positive vessels for control and TGN-020-treated animals, as well as the average number of stained vessels. Although the average diameter of IgG-positive blood vessels tended to be higher in all brain areas for control animals, this difference attained statistical significance only for the cerebellum (15.452 ± 3.326 μm vs 24.963 ± 2.210 μm, *P* = 0.0045), and from here for all the vessels pooled together (18.554 ± 3.255 μm vs 24.317 ± 1.941 μm, *P* = 0.0216) (Fig. 4, g). Regarding the number of stained vessels, control animals tended to show higher average values in all analyzed regions, except for basal ganglia, and with a significant difference only for the cortical vessels (5.632 ± 1.474 vs 7.822 ± 1.078 vessels/× 20 objective area, *P* = 0.0415) (Fig. 4, h).

### TGN-020 Does Not Induce Any Significant Variation in the Thickness of the Vascular Basement Membranes

**Fig. 5:**
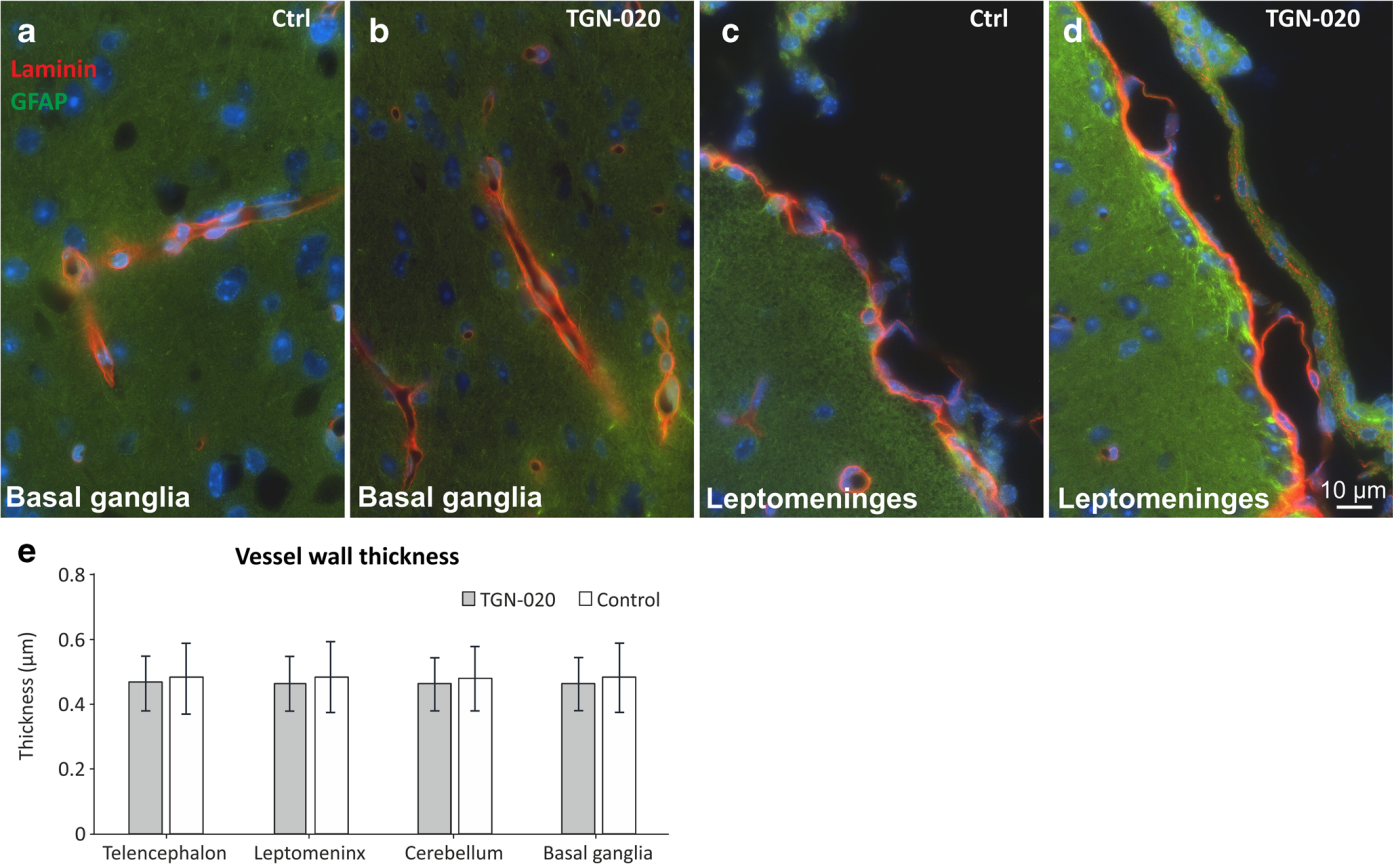
Post-fixed tissues immunostained for laminin and GFAP show no obvious differences between the basement membranes’ thickness utilizing light microscopy, nor any gliotic reaction, between the control and TGN-020-treated animals (**a**–**e**). The average value per animal was considered for analysis. Mean ± SD. *N* = 5 animals for the TGN-020 group and 3 animals for the control group

Next, we assessed the thickness of the vascular BM on × 60 deconvoluted fluorescence images captured for laminin and GFAP double stainings (Fig. 5, a–d). For all anatomical regions, and for both animal groups, there was a good homogeneity of BM thickness with basically no significant difference or even tendency, suggesting no major direct effect of TGN-020 (Fig. 5, e). Also, the GFAP staining did not show any differences in astrocytic density or preponderance for both animal groups. Lastly, we assessed whether there was a correlation between the vessel diameter and its measured BM thickness. Descriptive analysis revealed that in control animals, the blood vessel diameters showed a modest correlation with the wall thickness [*r*(374) = 0.262, *P* < 0.001], while this correlation was slightly lower in TGN-020-treated animals [*r*(414) = 0.113, *P* = 0.011].

### No Ultrastructural BBB Changes After TGN-020 Treatment

**Fig. 6:**
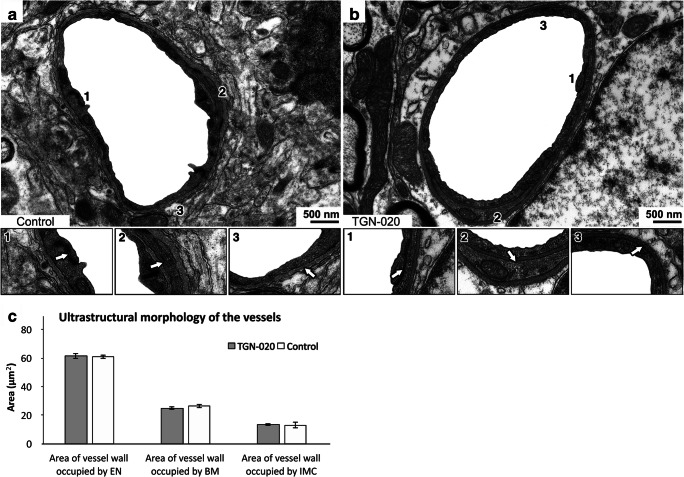
Inhibition of AQP4 by TGN-020 does not alter the structural appearance of the capillary vascular wall in the grey matter. There was no difference in the ultrastructure of cerebral capillaries from mice treated with TGN-020 (**b**) when compared with control mice (**a**). Endothelial tight junctions (a1 and b1), intramural cells (a2 and b2), and basement membranes (a3 and b3) appeared normal. The average value per animal was considered for analysis. Mean ± SD. *N* = 3 animals each of the control and TGN-020 groups

Qualitative analysis by TEM revealed no structural differences in the appearance of capillaries from grey matter (*n* = 60) of TGN-020-treated C57BL6 mice, compared with the same number of cortical vessels from control C57BL6 mice (Fig. 6, a–c). Specifically, a univariate analysis of variance (ANOVA) for TGN-020-treated and control animals revealed no significant differences in the percentage surface area of the vessel walls occupied by the endothelium (61.11% vs. 62.01%, *P* = 0.494), basement membrane (25.84% vs. 25.83%, *P* = 0.987), or intramural cells (13.05% vs. 12.16%, *P* = 0.492). These data suggest that a single massive dose of TGN-020 does not alter the ultrastructural structure of blood vessels.

## Discussion

AQPs form highly conserved water membrane channels in most living organisms, controlling osmotic-driven water movement around the cellular membranes in bacteria, plants, and mammals [43]. In humans, 13 distinct AQPs have been described, and 3 exist in the human CNS: AQP4, AQP1, and AQP9 [44, 45]. AQP1 has been described in the brain classically in the epithelia of the choroid plexus, at low levels in the endothelia, and also in the astrocytic membranes, where most of it was colocalized with AQP4 [44, 46, 47], while AQP9 seems to be restricted to substantia nigra [48].

To date, numerous Aβ clearing mechanisms have been described, all overlapping to different extents depending on their histological relationships to local cellular and vascular elements, but with unknown exact relative contribution towards the catabolism of total Aβ bulk and its subspecies. Intracerebral injection of different Aβ isoforms revealed that these enzymatic pathways act mostly on Aβ42 species, with the main enzyme, nepriliysin, being incapable of degrading Aβ40 [49]. As blood vessels collect most of the metabolic waste in any organ, these have also been implicated in several mechanisms of Aβ clearance. A trans-endothelial bi-directional pathway has been showed to be mediated by the low-density lipoprotein receptor-related protein (LRP) [50] and the receptor for non-enzymatic glycoxidation (RAGE) [51], with both transporters being reported again to be mediating mostly Aβ40 across the BBB. Besides trans-endothelial transport, a complex “glymphatic” pathway has been introduced to describe the fluid flow out of the brain [52]. Thus, the CSF flows in brain following arterial pulsations, between the basement membrane of the vascular smooth muscle cells and pia mater (Virchow-Robin perivascular space) and is transported across the astrocytic perivascular end-feet to enter interstitial fluid (ISF) of the neuropil. ISF then drains back into the CSF, either along the Virchow-Robin perivascular space or along the vascular basement membranes between the smooth muscle cells and the endothelial cells, but in both cases, it needs to cross through the astrocytic end-feet [31]. In the astrocyte end-feet, AQP4 is the main water channel that drives the bidirectional fluid flux around the bleed vessels [53, 54]. We have previously shown that a single administration of the TGN-020 AQP4 inhibitor on an animal model of ischemic stroke reduces brain cytotoxic oedema by limiting the plasma influx into the parenchyma, and that this effect is associated with thicker vascular basement membranes in ischemic animals treated with TGN-020 [27]. Moreover, there was a clear-cut colocalization of the vascular BM’s laminin with endogenous murine albumin in the walls of the vessels of these animals, this being a first direct proof that AQP4 inhibition decreases fluid passage around BBB at the level of the outer vascular basement membranes [27]. More recent data utilizing the AQP4 facilitator TGN-073 showed an increased fluid turnover at the level of the BBB, promoting circulation and clearance of the interstitial fluid along the BBB [55]. Also, the observation that intraparenchymal injection of ovalalbumin and dextran also leads to their drainage alongside the vascular basement membranes [56] consolidates this route as a general fluid drainage pathway, not specific but essential for the clearance of soluble Aβ pool.

In the present study, we have showed for the first time that AQP4 inhibition directly decreases soluble amyloid Aβ drainage through the intramural vascular pathways, suggesting that its downregulation at the level of the perivascular astrocytic end-feet, age-related or otherwise, would greatly contribute to the accumulation of Aβ in the brains of LOAD patients. Indeed, a number of astrocytic changes have been described in AD mouse models and human pathology, like detachment of astrocyte end-feet from the BBB, reduction of end-feet metabolism including loss of astrocytic glucose transporter 1 and of AQP4, and an overall impaired perivascular drainage of solutes [30, 57, 58]. Our experimental setup showed significant differences in Aβ vascular intramural retention between the TGN-020-treated/control animals, and this is despite the fact that surgically opening the skull reduces arterial pulsations and glymphatic flow [59]. Our in vivo results showed that for both inhibitor dosages, fluorescent Aβ showed a tendency to accumulate mostly in smaller diameter vessels for TGN-020-treated animals compared with the control group (*P* = 0.066). As the BBB per se exists only at the level of the capillaries, and AQP4 is mostly expressed in the perivascular glia limitans and is polarized towards the vascular basement membranes, it is conceivable that these sites are also the most susceptible of being affected by TGN-020. In line with our findings, tracer experiments and two-photon microscopy imaging on mice lacking AQP4 showed a 70% reduction in clearance of the interstitial fluid along the paravenous drainage pathways, suggesting that astrocyte end-feet is responsible for gating these clearance pathways [60]. This study [60] also assessed the paravascular drainage of Aβ40, although the injection was done in striatum, and assessment was on post-mortem vibratome sections. It showed that Aβ40 was cleared mainly along these pathways in wild-type animals, but in AQP4 knockouts, this drainage was abolished. Although the histology of the arteries in the basal ganglia is slightly different from the cortical arteries, as the former are coated by not one, but two distinct layers of leptomeninges [61], the diffusion principles that apply should be the same as for the cortical vessels. Moreover, AQP4 null mice crossbred with APP/PS1 mice not only show an increase in the total Aβ disposition in their brains compared with APP/PS1 animals but also an increase in the vascular-deposited Aβ (CAA) [62].

Also, we have showed here that the single massive dose of TGN-020 did not induce any detectable microscopical and ultrastructural changes at the level of the blood vessels or their basement membranes, and only to regional differences towards their permeability for endogenous murine immunoglobulins. Despite the fact the BBB comprises a non-fenestrated layer of endothelial cells bound by tight junctions, on a continuous endothelial basement membrane, its impermeability to serum components is not absolute. Under normal conditions, intravenous administration of human IgG in mice revealed that a fraction of the perfusate is able to cross the BBB without inflammation or without causing leakage [63]. In this direction, a variety of mechanisms have been described that might be able to facilitate the access of IgG in the parenchyma through the blood vessel wall, like for example binding to the neonatal Fc receptor (FcRn) [64], the LRP-1 [65], transferrin, or insulin receptors [66]. After adhesion, mechanisms like transcytosis and lysosomal degradation have been described to be involved in maintaining an equilibrium in the permeability/impermeability balance, and in fact impaired lysosomal degradation has been showed to be followed by IgG accumulation in the basement membranes [67]. Altogether, a combination of enhanced vascular permeability due to hypoxic conditions during animal euthanasia, together with the blockade of fluid at the basement membrane-glia limitans interface in TGN-020-treated animals, might explain the increased overall vascular permeability for endogenous IgG in control animals compared with the treated group. It can very well be that the high sensibility of cerebellum to hypoxia, as compared with the rest of the brain [68], together with the more complex anatomy of the vessels in the basal ganglia, surrounded by two distinct layers of leptomeninges separated by a perivascular space [61], could explain the differences observed in cerebellum and basal ganglia in our experiment. Vessel wall thickness was not deemed significantly different between control and TGN-020 animal groups, and there were only low correlation values between the diameters and wall thickness.

A massive TGN-020 administration is not accompanied by detectable functional and morphological changes at the level of the brain or other organs, or by any changes in the AQP4 expression patterns, but with immediate reduction of perivascular fluid exchange and of cytotoxic oedema [26, 27, 69]. Given the fact that PET-CT analysis of the presence of [11C]TGN-020 in the human brain revealed a first pass-effect within the first 10 min, followed by plateau levels for the brain parenchyma in the frame time of 15–60 min post-injection [69], all our experimental approaches (i.e. in vivo, ex vivo, and EM) fall within the same window of comparable functional and morphological changes. The advantage of pharmacological transient inhibition versus gene deletion is that for short period of time, compensatory mechanisms, like the coexistence of AQP1 in the brain, might temporarily take over for AQP4 loss, while AQP4 null animals have been reported to exhibit deficits of membrane potential formation in retinal Müller cells, impaired hearing, and memory consolidation [70–72]. The fact that AQP4 null animals show subtle metabolic changes, and putatively some other non-explored deficits, makes our experiment the most direct proof of the relationship between AQP4 inhibition and increased Aβ burden in the CNS and in the perivascular sector.

Although our work is a continuation of previous studies that have already shown that soluble Aβ injected into the brain parenchyma is drained according to the diffusion gradients through the capillary basement membranes, and within the basement membranes of the tunica media of cerebral arteries, but not associated with the veins [31, 56], the major drawback of the present study was that we could not differentiate in vivo the arterial from the venous sector, thus not being able to rule out the paravascular drainage pathways. However, to our knowledge, this is the first study that utilizes a transient AQP4 inhibition to observe the immediate in vivo behavior of soluble and diffusible Aβ40 in relationship to blood vessels.

Interestingly, Aβ accumulation in our in vivo experiment tended to occur especially in smaller diameter vessels after AQP4 inhibition, and IgG infiltration on fixed tissue was also identified in smaller diameter vessels. Both point to the fact that the first nidi of Aβ during plaque formation occurs in the BBB associated with smaller vessels, where the ISF drains directly from within the parenchyma [73]. CAA can be present without concurrent AD neuropathology; however, it has been showed to be present in up to 90% of the AD cases [74]. Moreover, not all CAA, but capillary CAA and small vessel pathology (arteriosclerosis), seems to be associated with allocortical microinfarcts and cognitive decline in AD patients [75, 76], so that it can be that Aβ disposition in the small vessels represents the first subtle change that precedes plaque formation that initiates the pathological/cognitive decline.

## Conclusion

This is the first direct proof that transient AQP4 inhibition rapidly reduces the intramural vascular drainage of the ISF from the brain, with a direct effect towards favoring the disposition of amyloid Aβ in the blood vessel walls. Taken together with the age-associated small vessel disease that occurs in AD patients, AQP4 function assessment and facilitation might be a good prognostic or treatment candidate in these patients.

## Electronic Supplementary Material

Supplementary Figure 1 Line profile pixel intensity analysis can differentiate Aβ40 positive vessels from Aβ40 negative vessels even on (A) a high-background image from TGN-020 treated animals (>0.5 μl volume group). A line of 100 μm is placed across selected vessels (yellow for negative vessels, and white for positive vessels). (B) A histogram of one of the vessels shows the intensity profiles for the red-green channels along the line, and arrowheads denotes the edge between SR 101 and parenchymal Aβ40, and are considered at 0 μm point from where the intensity of the green pixels are analyzed (C, D). The average green pixel intensity in the first 3 μm around the lumen of the vessel is higher than that for the next 7 μm only for the positive vessels group. Mean ± SD. ∗∗*P* < 0.01. *N* = 16 measurements for each type of vessel (positive / negative) (8 vessels per type are quantified on both sides of their lumen). (PNG 4839 kb)

High resolution image (TIF 20695 kb)

Supplementary Figure 2 Five minutes-time steps of two-photon microscopy images for the control and TGN-020 treated animals injected with either low (<0.5 μl) or high (<0.5 μl) volumes of Aβ40-A488. The tracer takes longer to be completely drained from the parenchyma and shows a more frequent peri-vascular disposition in TGN-020 treated animals; arrows indicating a p denominate the position of the injection pipette tip, filled with the fluorescent Aβ40. (PNG 25347 kb)

High resolution image (TIF 129442 kb)

ESM 1 (DOCX 14 kb) (DOCX 14 kb)

## Data Availability

All raw data is available from the corresponding authors upon reasonable request.
