# Supplementary figures and images for: Inhibition of Aquaporin 4 Decreases Amyloid Aβ40 Drainage Around Cerebral Vessels
Source: Mol Neurobiol. 2020 Aug 11;57(11):4720–34. doi: 10.1007/s12035-020-02044-8 (PMC7515968; doi:10.1007/s12035-020-02044-8)

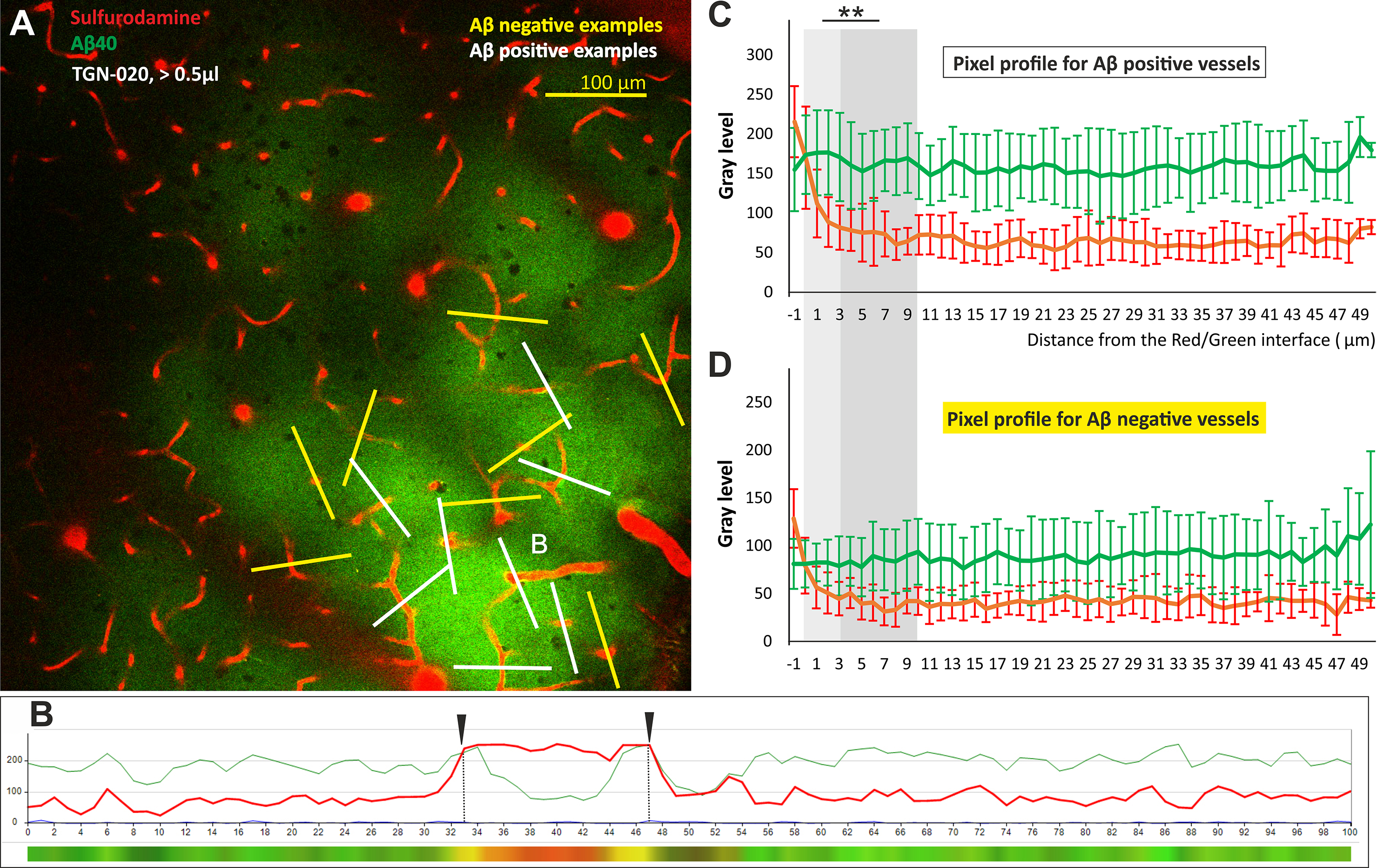

Supplement: Supplementary file 1 — Line profile pixel intensity analysis can differentiate Aβ40 positive vessels from Aβ40 negative vessels even on (A) a high-background image from TGN-020 treated animals (>0.5 μl volume group). A line of 100 μm is placed across selected vessels (yellow for negative vessels, and white for positive vessels). (B) A histogram of one of the vessels shows the intensity profiles for the red-green channels along the line, and arrowheads denotes the edge between SR 101 and parenchymal Aβ40, and are considered at 0 μm point from where the intensity of the green pixels are analyzed (C, D). The average green pixel intensity in the first 3 μm around the lumen of the vessel is higher than that for the next 7 μm only for the positive vessels group. Mean ± SD. ∗∗P < 0.01. N = 16 measurements for each type of vessel (positive / negative) (8 vessels per type are quantified on both sides of their lumen). (PNG 4839 kb) [file 12035_2020_2044_Fig7_ESM.png]

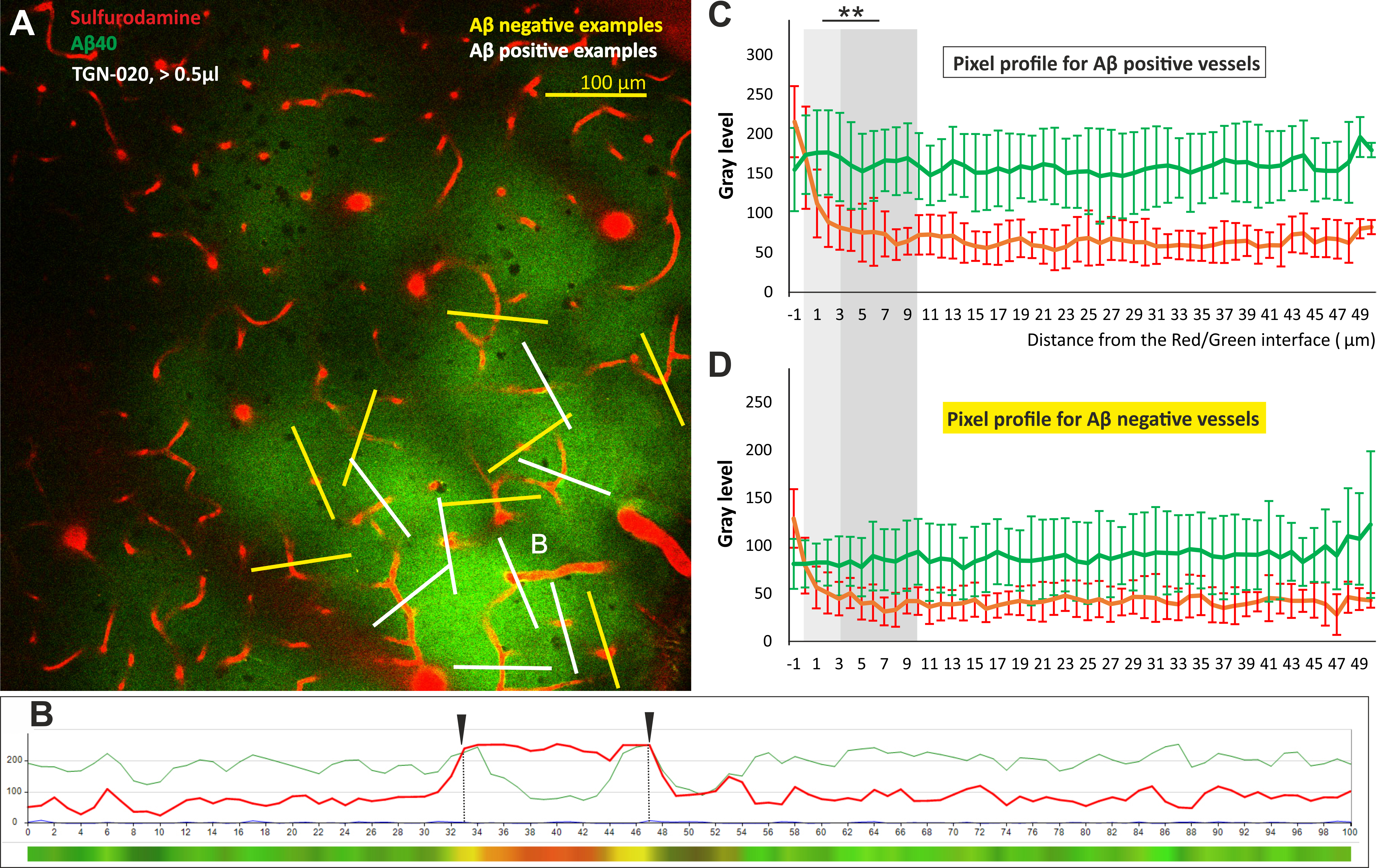

Supplement: Supplementary file 2 — High resolution image (TIF 20695 kb) [file 12035_2020_2044_MOESM1_ESM.tif]

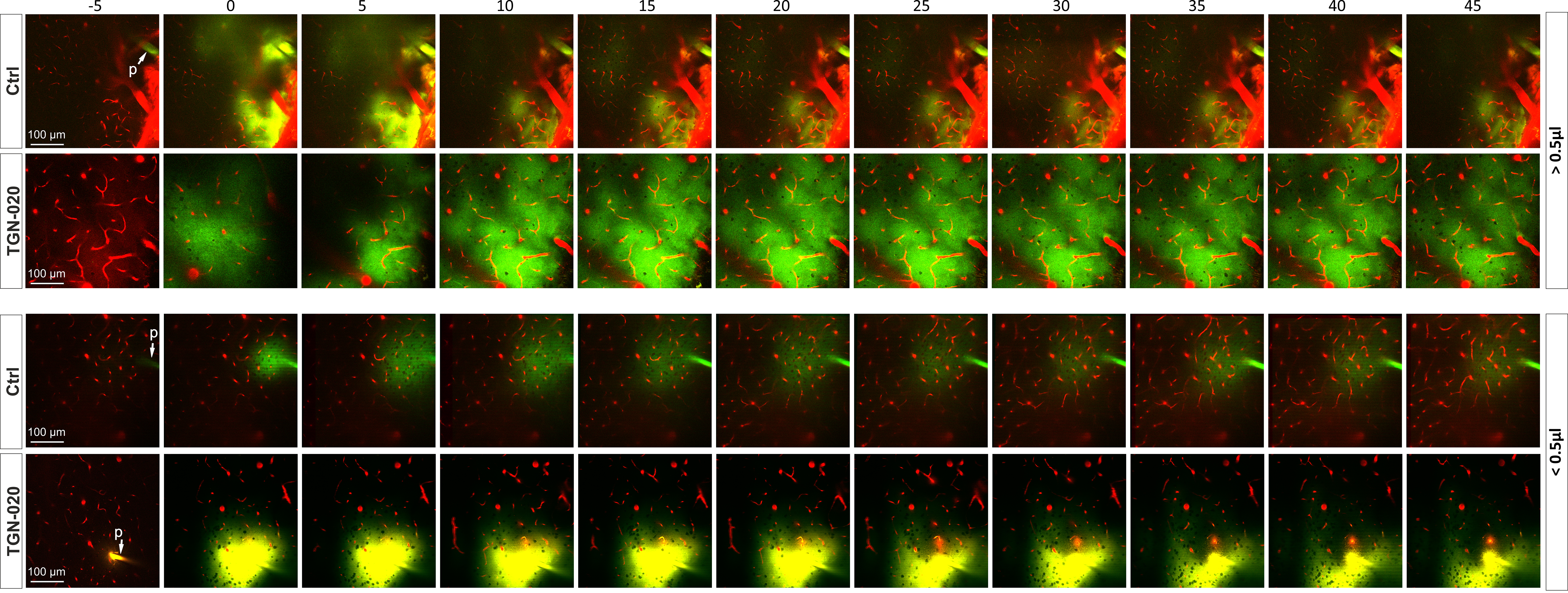

Supplement: Supplementary file 3 — Five minutes-time steps of two-photon microscopy images for the control and TGN-020 treated animals injected with either low (<0.5 μl) or high (<0.5 μl) volumes of Aβ40-A488. The tracer takes longer to be completely drained from the parenchyma and shows a more frequent peri-vascular disposition in TGN-020 treated animals; arrows indicating a p denominate the position of the injection pipette tip, filled with the fluorescent Aβ40. (PNG 25347 kb) [file 12035_2020_2044_Fig8_ESM.png]
