# Supplementary material for: Inhibition of Aquaporin 4 Decreases Amyloid Aβ40 Drainage Around Cerebral Vessels
Source: Mol Neurobiol. 2020 Aug 11;57(11):4720–34. doi: 10.1007/s12035-020-02044-8 (PMC7515968; doi:10.1007/s12035-020-02044-8)
Supplement: Supplementary file 5 — (DOCX 14 kb) (DOCX 14 kb) [file 12035_2020_2044_MOESM3_ESM.docx]

# Gabriela-Camelia Rosu et al. **Supplementary material**

# **Supplementary Materials and Methods**

In order to see if a machine learning algorithm might differentiate between what we considered Aβ40 positive / negative vessels in our analysis, we have selected one exemplary image where we considered all positive vessels in one category (N=8), and an equal number of negative vessels in the second category. Moreover, we choose the image in such a way that both type of vessels to be surrounded by a high level of diffused Aβ signal to make the differentiation more difficult (**Supplementary Fig 1**).

In the demonstration version of Image ProPlus 10 package, we have utilised the line profile intensity tool which can deliver grey value pixel intensities along a predefined line as a function of distance along the line from its origin. A line of 100 µm was manually arranged repetitively on different vessels, through the middle of the lumen (for transversally sectioned vessels), or perpendicular to the axis, for longitudinally sectioned vessels. For every vessel, the pixel intensity profile was saved in an Excel sheet, noting the two middle coordinates where the green and red plots intersected (the edge between Sulfurodamine 101 and Aβ40 green signal). These coordinates where considered the 0 µm distance value from the vessel lumen, and all intensities along the lines were furthered referred to this origin for each side of the vessel (thus resulting in 16 measurements for 8 vessels). All data for each group of vessels (positive or negative) has been aligned to the origin, averaged, and then for each micron away from the origin we plotted pixel intensities. In order to asses if the green channel contained more signal in the immediate vascular vicinity, we have considered all the data until 3 µm away from the vessel wall, and compared it with the data from the 3-10 µm interval.

# **Supplementary results**

Our objective proof-of principle analysis revealed a peak in the green pixel intensities in the immediate vicinity of the blood vessels for the group of positive vessels, and a more homogenous distribution for the Aβ negative group (**Supplementary Fig 1**). Also, there was a higher level of background for the Aβ40 positive group. The average values of the green pixel intensities were significantly higher for the first 3 µm around the vessel interface, compared to the next 7 µm for the positive vessels (*P*=0.004), but not for the negative vessels (*P*= 0.177).
